# Implementing LGBTQ-affirmative CBT: study protocol for an effectiveness-implementation trial at 90 LGBTQ community centers

**DOI:** 10.1186/s12913-025-13136-3

**Published:** 2025-07-21

**Authors:** John E. Pachankis, Danielle Chiaramonte, Hunter T. Baldwin, Corey Prachniak, Deborah S. Levine, Shawn Van, Rebekah J. Hobbs, Hadley Ankrum, Lauren J. Wilkins, Tyler D. Harvey, Audrey Harkness, Skyler D. Jackson, Em Matsuno, Zachary A. Soulliard, Briana S. Last, Julian Burger, Molly Delehant, Xin Zhou, Ashley K. Hagaman, Dennis H. Li, Brian Mustanski

**Affiliations:** 1 Department of Social and Behavioral Sciences, Yale School of Public Health, New Haven, CT USA; 2 CenterLink: The Community of LGBTQ Centers, Fort Lauderdale, FL USA; 3 School of Nursing and Health Studies, University of Miami, Miami, FL USA; 4 Department of Psychology, Arizona State University, Tempe, AZ USA; 5 Department of Psychology, Miami University, Oxford, OH USA; 6 Department of Psychology, Stony Brook University, Stony Brook, NY USA; 7 Department of Medical Social Sciences, Feinberg School of Medicine, Northwestern University, Chicago, IL USA; 8 Department of Psychiatry and Behavioral Sciences, Feinberg School of Medicine, Northwestern University, Chicago, IL USA

**Keywords:** Implementation science, Mental health, Sexual and gender minority, Cognitive-behavioral therapy, Stigma

## Abstract

**Background:**

Sexual and gender minorities (SGM) experience among the largest mental health disparities of any population. One driver has been the lack of evidence-based practices (EBPs) addressing the distinct mechanisms underlying SGM’s risk. LGBTQ-affirmative cognitive-behavioral therapy (CBT) is among the only EBPs specifically for SGM mental health. LGBTQ community centers represent an ideal implementation setting for LGBTQ-affirmative CBT given their wide reach. Although direct training of mental health providers at LGBTQ community centers by experts has been shown to improve providers’ LGBTQ-affirmative CBT skills, it is unclear how such training should be optimally delivered. This paper describes the protocol of a trial that seeks to compare the effectiveness of three training strategies for implementing LGBTQ-affirmative CBT, identify center-level moderators of implementation success, and examine the impact of the three strategies on client mental health through theory-informed organizational and provider mechanisms.

**Methods:**

This hybrid type III effectiveness-implementation trial will randomize 90 centers to receive one of three additive strategies for implementing LGBTQ-affirmative CBT: [1] a suite of self-paced digital learning materials (Materials Only condition); [2] these materials plus weekly live webinar training for 12 weeks (Direct Training condition); or [3] the above plus one year of supervision from a local supervisor who will receive expert consultation in a train-the-trainer format (Local Supervision condition). The primary outcome will be provider fidelity assessed via simulated practice. Implementation determinants (e.g., center resources, provider/client characteristics), mediators (e.g., implementation climate, provider self-efficacy), and other outcomes (e.g., intervention adaptation, sustainment) will be captured using a mixed-methods design. Clinical effectiveness outcomes (i.e., client mental and behavioral health symptoms) will be assessed through client surveys among a subset of 15 centers.

**Discussion:**

Now that LGBTQ-affirmative CBT has shown efficacy across several trials and generated high demand, research is needed to determine nationwide implementation strategies. This study will identify optimal means through which to implement this treatment innovation in the US’s large network of LGBTQ community centers, thereby producing generalizable guidance for EBP implementation across low-resource settings nationwide in which mental health disparities populations are likely to seek treatment.

**Trial registration:**

NCT05890404 (05/25/2023), https://clinicaltrials.gov/study/NCT05890404.

**Supplementary Information:**

The online version contains supplementary material available at 10.1186/s12913-025-13136-3.

## Background

Sexual and gender minority (SGM) individuals experience a significantly elevated risk of adverse mental health conditions; specifically, consistent evidence suggests that SGM individuals are at a 1.5-4 times greater risk for depression, anxiety, substance use problems, and suicidality compared to the general population [1–6]. These disparities are driven by LGBTQ-related stigma [2, 7] from the broadest structures (e.g., laws and policies denying, or failing to protect, SGM equality [8]) to daily events (e.g., bullying, family rejection [9–11]). This stigma gives rise to LGBTQ-related stress reactions, at least partially explaining SGM individuals’ elevated risk of poor mental health [12–16]. These stress reactions include cognitive (e.g., hypervigilance), affective (e.g., shame), and behavioral (e.g., concealment) processes [17, 18]. Bisexual, transgender, and racial/ethnic minority SGM are at particular risk for mental health challenges due to intersectional forms of stigma [19–22].

SGM individuals experience several barriers to accessing affirmative mental health care that is responsive to stigma-related stress reactions, thereby perpetuating the disparity [23–26]. For instance, SGM individuals report shame in discussing their stigma experiences and often mistrust mental health providers, perhaps because of the historical anti-SGM stance of the mental health field, the continued practice of harmful so-called “conversion therapies,” and mental health providers’ role as gatekeepers of some forms of care [27–33]. The lack of evidence-based practices (EBPs) — practices supported by research evidence — that affirmatively address SGM individuals’ distinct stigma-related concerns have also historically impeded SGM people’s treatment access, further perpetuated by a lack of available mental health providers trained in affirmative treatment approaches [31, 34].

LGBTQ-affirmative cognitive-behavioral therapy (CBT) was developed to bring scientific evidence and stigma theory to bear on SGM people’s mental health care [35, 36]. It is one of the first mental health treatments with efficacy drawn from clinical trials showing evidence for improving SGM mental health [37, 38]. LGBTQ-affirmative CBT contains nine modules that focus on minority stress awareness, strategies for challenging internalized stigma, emotion awareness and regulation, and behavioral skills to mitigate minority stress reactions [39, 40]. The treatment encourages providers to create a case conceptualization that considers the early and ongoing role of minority stress in each client’s current distress [41]. Typically administered individually by a trained mental health provider, the treatment can be delivered in as few as ten sessions or extended based on clinician judgement. LGBTQ-affirmative CBT is particularly suited to addressing SGM’s mental health given that it identifies social determinants of mental health (rather than internal deficits), facilitates insight into internalized stigma (rather than questioning the existence of stigma), and promotes coping self-efficacy [42]. LGBTQ-affirmative CBT also lends itself to implementation in frontline settings given that it is skills based, present focused, and time limited [43–47].

The efficacy of LGBTQ-affirmative CBT has been tested across several randomized controlled trials with various SGM populations. In two early waitlist trials (*n* = 63 young sexual minority men; *n* = 60 young gender-diverse sexual minority women), LGBTQ-affirmative CBT yielded moderate-to-large reductions in depression, anxiety, and alcohol use [37, 38]. These trials also found significant engagement of the hypothesized target mechanisms: internalized homonegativity, social isolation, and emotion dysregulation. In a larger trial (*n* = 254 sexual minority men), LGBTQ-affirmative CBT was particularly efficacious in reducing substance use problems and mental health comorbidity compared to two control conditions– treatment-as-usual and screening/brief intervention/referral [48]. Moderator analyses showed that LGBTQ-affirmative CBT was most efficacious in addressing comorbid depression, anxiety, substance use problems, and HIV-transmission-risk behavior among Black and Latinx men [49]. Additional trials have found support for LGBTQ-affirmative CBT’s initial effectiveness and strong acceptability among Black and Latinx sexual minority men [50], sexual minority young men in China [51], and transgender and gender-nonbinary individuals [52].

Few nationwide settings exist for studying the implementation of mental health innovations like LGBTQ-affirmative CBT. However, the US has over 300 LGBTQ community centers that meet the SGM community’s otherwise-unaddressed social, legal, and health needs. LGBTQ community centers are those with a primary goal to support the LGBTQ community by offering formal programming, from support groups to comprehensive health care [53, 54]. Given their role in serving over 48,000 clients/year, most of whom are Black and Latinx and earn <$30,000/year [55], LGBTQ community centers represent a promising venue in which to address the disproportionate mental health challenges affecting SGM individuals by training providers to deliver LGBTQ-affirmative CBT. Our team prepared for the implementation of this treatment in LGBTQ community centers through stakeholder meetings [56], center director surveys [56], a pilot training study [57], and interviews with implementers [58]. Through this work, we learned that training providers at LGBTQ community centers in LGBTQ-affirmative CBT is feasible, acceptable, and efficacious. Center directors strongly endorsed the need for their mental health providers to be trained in LGBTQ-affirmative CBT and unanimously supported allowing staff time for this [56].

In a waitlist trial across 50 centers, mental health providers (*n* = 120) rated a 12-week (one hour per week) training in LGBTQ-affirmative CBT to be informative and motivating, and results showed that the training improved their competence, knowledge, and skills in LGBTQ-affirmative CBT [57]. Other data show that mental health providers in clinical trials of LGBTQ-affirmative CBT have rated ongoing supervision as the most effective implementation support [58]. Although EBP training and supervision is generally effective, recent systematic reviews identify three knowledge gaps for the field of EBP training overall [59–61], including: (1) whether train-the-trainer strategies can sustain EBP implementation beyond any initial training; (2) whether features of resource-constrained settings, characterizing many LGBTQ community centers (e.g., high staff turnover), predict the optimal EBP implementation strategy needed; and (3) the target mechanisms through which EBP training impacts implementation *and* client outcomes.

To date, LGBTQ-affirmative CBT has only been tested in controlled trials in research settings [37, 38, 48, 49, 62, 63]. At the same time, mental health providers working in real-world settings (e.g., Veterans Health Administration, college counseling centers, community mental health) have begun implementing the treatment without evaluation since the publication of the treatment manuals [39, 40]. While this reflects the high demand for this treatment, it has not produced generalizable knowledge about implementation. In this current trial, we draw on two implementation frameworks, Reach, Effectiveness, Adoption, Implementation, and Maintenance (RE-AIM) [64] and the Consolidated Framework for Implementation Research (CFIR) [65, 66], to conduct a type III hybrid effectiveness-implementation trial to test LGBTQ-affirmative CBT’s real-world effectiveness and implementation outcomes across three additive training conditions — i.e., the three implementation strategies tested in this trial: (1) a self-paced suite of digital learning materials, including provider guides and training videos (Materials Only condition); (2) these material plus a weekly live webinar training for 12 weeks (Direct Training condition), or (3) the above plus supervision from a local supervisor who themselves will receive expert consultation in a train-the-trainer format (Local Supervision condition).

**Fig. 1:**
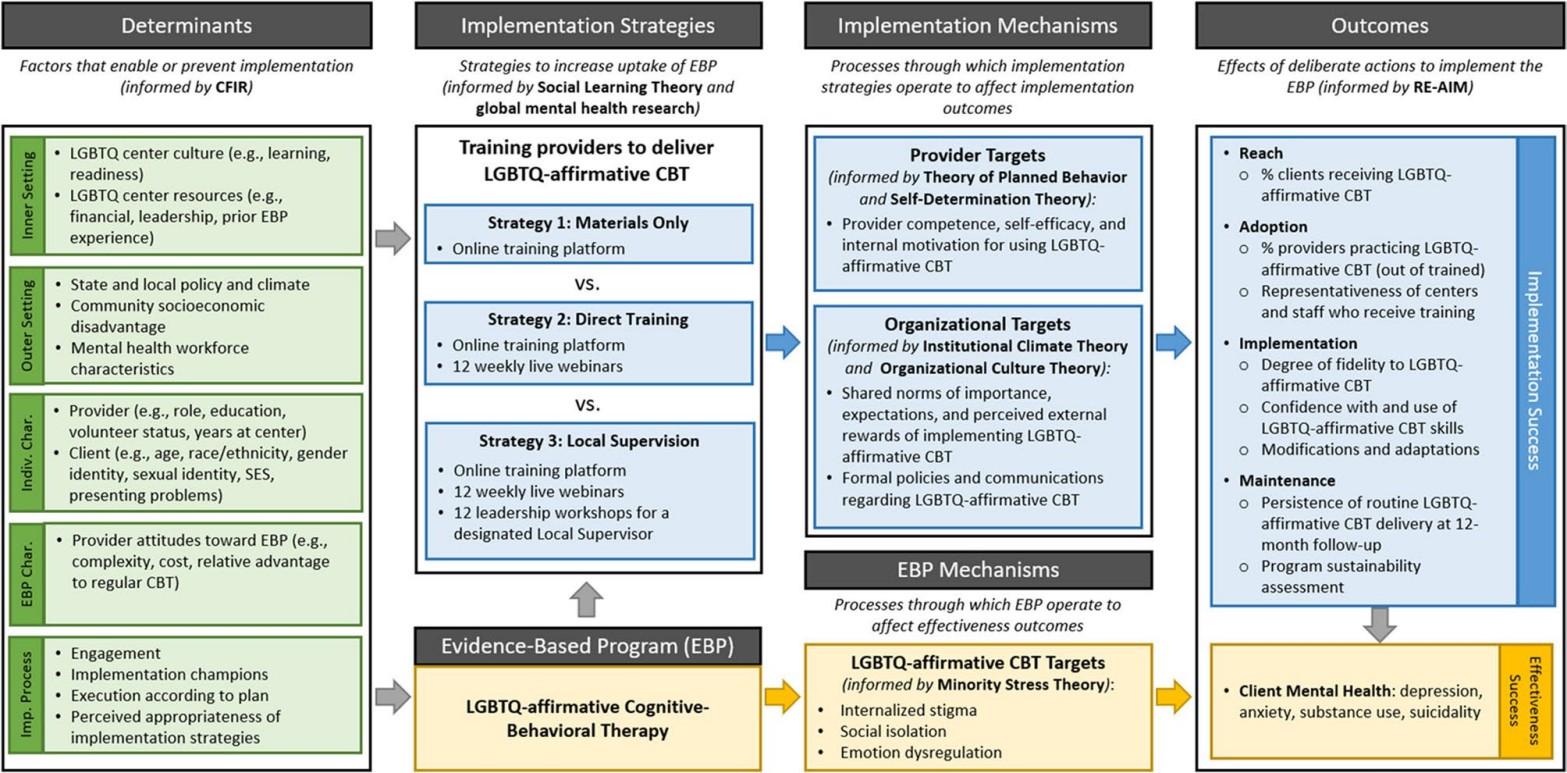
Implementation research logic model for comparing three training strategies to enhance the implementation of LGBTQ-affirmative CBT in LGBTQ community centers

We hypothesize that these three additive training implementation strategies will predict respective graduated increases in implementation success at 4-, 8-, 12-, and 24-months post-training (see Figs. 1 and [67]). That is, we predict that reach, effectiveness, adoption, implementation, and maintenance will be higher in the Local Supervision condition relative to the Direct Training condition and that the Direct Training condition’s implementation outcomes will be higher than the Materials Only condition. Drawing on theories of organizational [68, 69] and provider [70–73] change, we also hypothesize that organizational (e.g., shared norms of the importance of EBP) and provider (e.g., self-efficacy for EBP delivery) factors will mediate the stronger impact of the more intensive training strategies on implementation success and client mental health outcomes. To facilitate this latter aim, in a subset of 15 LGBTQ community centers (five centers per condition), we will compare the three training implementation strategies in terms of client mental health symptom change from before implementation to 4, 8, 12, and 24 months after.

## Methods

### Aims

This trial employs a type III hybrid effectiveness-implementation design to pursue the following three aims:

1. Compare the success of three additive LGBTQ-affirmative CBT implementation strategies (Materials Only, Direct Training, and Local Supervision) across 90 LGBTQ community centers.
2. Identify center-level determinants of successful implementation of LGBTQ-affirmative CBT.
3. Examine the impact of three implementation strategies on client mental health through theory-informed target mechanisms at both organizational and provider levels.

### Setting

We will implement one of these three LGBTQ-affirmative CBT training strategies at 90 LGBTQ community centers in the US. Of the more than 300 LGBTQ community centers in the US, about 200 provide mental health services [55, 56]. The 90 centers will be selected based on responsiveness and capacity (e.g., anticipating at least two providers who would participate in the training). The 90 included centers for this study will represent every region of the US, serve urban and rural areas, and represent the majority of US states. With small operating budgets, these centers are often staffed by mental health trainees and peer volunteers [55]. Further, most centers currently offer non-EBP supportive therapy [56]. Enhancing LGBTQ community centers’ capacity to implement LGBTQ-affirmative CBT would (1) bring EBP to thousands of SGM clients per year, (2) train early-career mental health providers to bring this EBP to their future work settings, and (3) identify the best strategies and organizational and provider targets for implementing EBPs for disparity populations in low-resource settings nationwide.

### Design

A type III hybrid effectiveness-implementation design has been selected given that efficacy for this treatment has already been established while real-world effectiveness has not [37, 38, 48, 51, 74, 75]. LGBTQ community centers will be randomly allocated to one of the three implementation strategies for the duration of the study. Because randomization will occur at the center level, mental health providers working in the same center will receive the same one of the three training strategies, each described in further detail below. This cluster-randomized design has been selected to imitate the real-world context of EBP implementation (that is, change in provider practice typically occurs at the organization-level) [76], promote equity across providers working at the same center, and accommodate EBP diffusion across providers working at the same center.

**Fig. 2:**
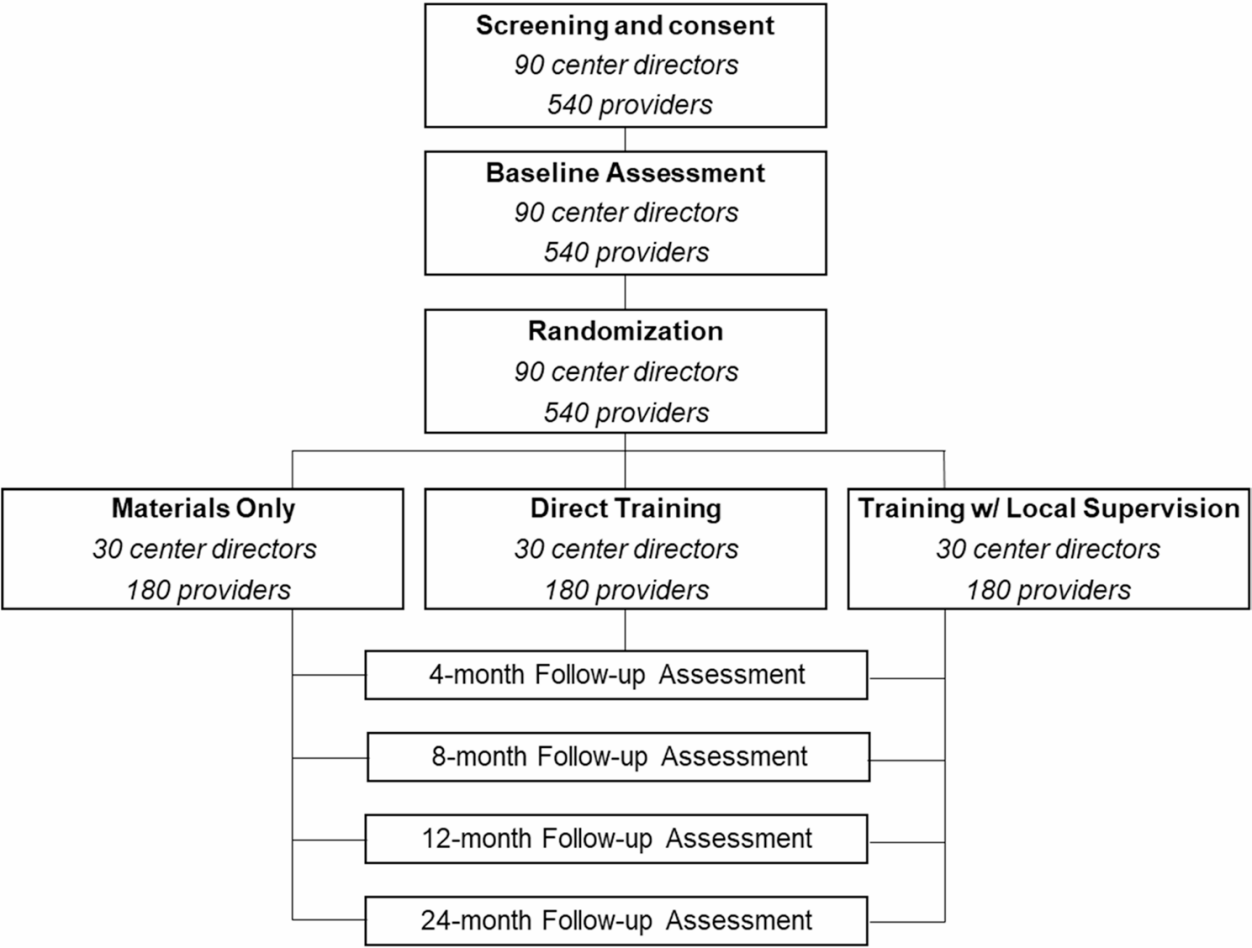
CONSORT: Study flow for mental health provider and director participants

**Fig. 3:**
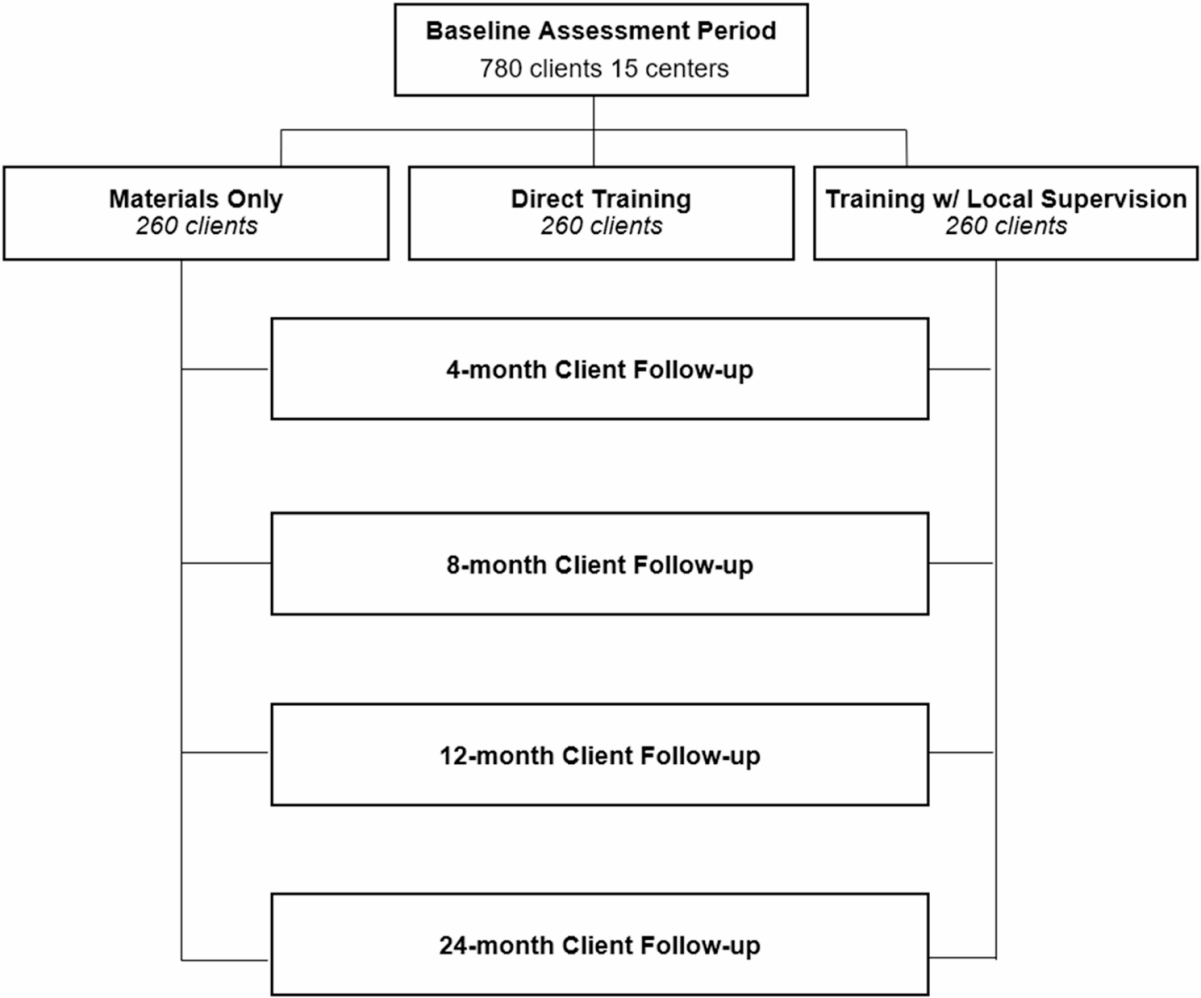
CONSORT: Study flow for client participants

To assess implementation outcomes, determinants, and mechanisms, we will collect survey data from mental health providers (*n* = 540), local supervisors (*n* = 30), and directors (*n* = 90) at baseline and 4-, 8-, 12-, and 24-month follow-ups. Interview data will be collected from providers, directors, and local supervisors throughout the follow-up period selected according to a sampling matrix designed to ensure equal distribution across geographic regions, center budget, and implementation condition among interviewees. The study flow for Aims 1 and 2 is illustrated in Fig. 2. To assess effectiveness outcomes (Aim 3), a subset of 15 centers have agreed to administer brief surveys with standardized symptom checklists and demographic questionnaires to their clients (*n* = 780) over the course of the study (see Fig. 3). Mental health providers, directors, local supervisors, and clients will be compensated for each survey and interview completed, and continuing education credits will be offered for each training module completed.

### Center enrollment

We have partnered with CenterLink, an international nonprofit organization and network of LGBTQ community centers. At least 200 CenterLink-affiliated centers provide mental health services, and others offer referrals to local mental health providers [55]. We will send targeted email communications to directors of these centers, informing them of this trial and training opportunity. Centers are eligible to enroll in this trial if they are (1) a current member of the CenterLink network, (2) based in the United States, and (3) anticipate at least two providers associated with their center participating in the training/study. Directors at centers that meet these criteria will sign a memorandum of understanding to officially enroll their center into the trial. We aim to enroll 90 centers.

In addition, 15 of the 90 enrolled centers will be selected to participate in Aim 3 of the study, in which they will additionally be asked to facilitate the collection of client mental health data at baseline and through the 24-month follow-up through activities such as distributing information about the survey to their mental health clients. These 15 centers were selected a priori to represent diversity across geographic region, budget, and populations served. Shortly before client data collection begins, these centers will be mailed palm cards containing client recruitment text alongside instructions regarding how to introduce the survey to clients.

### Randomization

Upon receiving 90 signed memoranda of understanding, the 90 LGBTQ community centers will be randomized in a 1:1:1 ratio to receive one of the three implementation strategies. Randomization will be stratified by director report of anticipated staff participation. Specifically, high-attendance centers will be operationalized as those with greater than the median number of anticipated providers across the 90 enrolled centers whereas low-attendance centers will be operationalized as those that anticipate fewer than the median number of providers per center. Centers whose anticipated number of providers is at the median will be randomly and evenly divided across the two groups. The 15 effectiveness centers will be randomized separately to ensure their equal distribution across the three implementation strategies. Randomization allocation will be generated by the study statistician and assigned by the study coordinator. All participants and study staff will be unmasked to condition assignment, as necessitated by the study design.

### Participants

This trial involves four classes of participants: directors (Aims 1–3), mental health providers (Aims 1–3), local supervisors (Aims 1–3), and clients (Aim 3).

#### Directors

Upon randomization, each of the 90 centers will be asked to nominate an individual to complete a director survey at baseline. Directors must be 18 years or older and in a clinical director, executive director, or similar leadership role at a participating LGBTQ community center. Directors may be double-enrolled as providers (i.e., participating in the training) and/or local supervisors and will be asked to complete survey questions for each of their roles at each time point. Directors will be contacted via email to complete follow-up [77] surveys at 4-, 8-, 12-, and 24-months post-baseline. In the event that a director leaves their position, they will be asked to nominate a new director at the center who will be invited to enroll in the study and complete all subsequent follow-up surveys.

#### Mental health providers

Following randomization, mental health providers working at or affiliated with enrolled LGBTQ community centers will be invited to register for the training. Eligible providers must (1) be 18 years or older; (2) provide or be planning to provide mental/behavioral health services (e.g., therapy, support groups, case management) to LGBTQ clients; and (3) be affiliated with an enrolled LGBTQ community center. In order to meet the third criterion, providers may either be employed or volunteer at a participating LGBTQ community center or be in the referral network of a participating LGBTQ community center (i.e., receive referrals or intending to receive referrals of mental health clients from the center). Mental health providers will be recruited by center leadership. Upon randomization, center leaders will distribute flyers to their employees, volunteers, and referral network providers to inform them of the study/training opportunity. Center directors will be asked to share information about the benefits of the study/training (e.g., continuing education credits, free training materials, financial incentive for assessments).

To be eligible to attend the training, mental health providers must provide consent and complete the baseline survey. Upon training launch, enrolled providers will be given instructions on how to access their respective training materials and, if applicable, live webinar training sessions. Regardless of their engagement with their respective training intervention or whether they remain affiliated with an LGBTQ community center, all providers will be contacted to complete follow-up surveys at 4-, 8-, 12-, and 24-months post-baseline. No concomitant interventions are prohibited for mental health providers enrolled in the trial.

#### Local supervisors

Upon randomization, centers randomized into the Local Supervision condition (described below) will be asked to nominate an individual to serve as the local supervisor, who will champion the supervision, training, and sustainment of LGBTQ-affirmative CBT at their center. The local supervisors should have significant therapeutic experience and be comfortable and interested in (1) learning how to oversee other providers at their center as they learn to deliver LGBTQ-affirmative CBT and (2) training future mental health providers to deliver LGBTQ-affirmative CBT. Local supervisors will be asked to attend an additional 12-week LGBTQ-affirmative CBT leadership workshop following the conclusion of the live webinar trainings, in addition to answering survey questions at all five assessment time points. To be eligible to attend the leadership workshop, local supervisors must provide consent and complete the baseline survey. Local supervisors may be simultaneously enrolled as directors and/or providers.

#### Clients

Clients will be eligible to take part in Aim 3 of the trial if they (1) are old enough to consent to behavioral health services in their respective state, (2) self-identify as a sexual and/or gender minority (e.g., LGBTQ), and (3) are currently or have recently received (within the past four months) mental/behavioral health services from one of the 15 effectiveness sites. Clients enrolled at the first time point will be asked to complete surveys at baseline and 4-, 8-, 12-, and 24-months post-baseline to assess short-term and long-term change in mental health outcomes. New clients will also be enrolled at all subsequent assessment time points.

### Implementation strategies

Based on a review of implementation strategies [77], our formative research with stakeholders, and the current state of EBPs for SGM mental health [77], we deemed training providers in LGBTQ-affirmative CBT to be the most feasible and significant way to sustain its implementation. We will test three training implementation strategies: (1) Materials Only, (2) Direct Training, and (3) Local Supervision. These conditions will be additive, so that each level also receives the supports of the lower levels.

#### Materials only

A digital suite of training resources has been shown to promote EBP implementation in resource-constrained settings [78]. We have developed an online training platform that contains 12 modules of highly engaging content, including (1) professionally filmed and edited instructional lectures featuring our team of expert trainers based on the published treatment manual [39], (2) instructional animations created by a professional animator detailing central concepts of the treatment, (3) video demonstrations of treatment delivery featuring our expert trainers and LGBTQ actors, (4) downloadable client worksheets, (5) optional readings to enhance learning, and (6) interactive exercises. This online suite contains approximately 10–12 h of content hosted on the Coursera learning platform and is designed to be completed at the user’s own pace. Regardless of study condition, all providers will receive access to the platform and a physical or digital copy of the treatment manual [39]; providers randomized to the Materials Only condition will only receive access to the platform and manual. Providers in all conditions will retain access to the online platform throughout the entire follow-up period. Participants will receive email reminders about the training. Engagement and adherence will be monitored.

#### Direct training

Direct training will consist of 12 weekly 60-minute live webinars on delivering LGBTQ-affirmative CBT led by a team of four expert trainers. Training will include didactics, “how-to” videos for each module, role-plays, opportunities for questions and answers, and experiential learning activities, as informed by social learning theory [79–83]. In designing this training, we considered sequence, spacing, modality, and duration of activities, as informed by adult learning theories [84]. A waitlist-controlled trial of this training showed efficacy across provider outcomes when compared to waitlist [57]. Providers will be able to attend one of two training session times each week. Providers in both the Direct Training and Local Supervision conditions will receive access to the live webinars, in addition to access to the online training platform and therapist manual. Participants will receive email reminders about the training. Engagement and adherence will be monitored weekly.

#### Local supervision

Four weeks after the final direct training session, the 30 centers randomized to the Local Supervision condition will send their nominated local supervisors to the first of 12 remote leadership workshop sessions focused on LGBTQ-affirmative supervision, training, and sustainment. In the train-the-trainer format to be employed in the Local Supervision condition, expert clinicians will provide additional training to local supervisors with the goal of increasing their ability to train other local mental health providers into the future. This training strategy has the benefit of harnessing local expertise, potentially making LGBTQ-affirmative CBT more responsive to local realities [85–87]. The goal of the LGBTQ-affirmative CBT leadership workshop is to provide local supervisors at LGBTQ centers with the tools, skills, and knowledge to become leaders and experts in LGBTQ-affirmative CBT and to train and supervise mental health providers at their center in LGBTQ-affirmative CBT for sustainment of this practice locally. Participants will receive email reminders about the training. Engagement and adherence will be monitored weekly.

## Outcomes

Guided by the RE-AIM framework, we will assess both implementation and effectiveness outcomes across relevant center, provider, and client levels quantitatively and qualitatively. Quantitative assessments will be captured through surveys at baseline and 4-, 8-, 12-, and 24-months post-baseline. Semi-structured interviews with select providers, directors, and local supervisors will be conducted throughout the follow-up period to provide additional context for understanding quantitative findings.

### Implementation outcomes

In Aim 1, we will assess the implementation success of three LGBTQ-affirmative CBT implementation strategies across the 90 LGBTQ community centers. The primary implementation outcome is provider fidelity to LGBTQ-affirmative CBT using our validated “simulated practice” assessment [64]. Providers will watch two brief (3-minute) video clips of an SGM actor roleplaying a presenting concern and then briefly describe in writing the clinical approaches they would use to address the concern. Research assistants will then code responses to assess participants’ application of LGBTQ-affirmative CBT skills using our reliable (ICC = 0.87) and valid rating scheme (57). Secondary implementation outcomes include mixed-methods assessments of the reach, adoption, familiarity, modification, and maintenance of LGBTQ-affirmative CBT skills across centers (See Table 1 for operationalizations).

**Table 1:** Measures

Outcomes	Measures	Data Source	Time point
Primary outcomes			
Implementation fidelity	∙ Simulated practice assessment [57] based on previous EBP research [117–120].	Provider simulated practice assessment	baseline, 4 m, 8 m, 12 m, 24 m
Secondary outcomes			
Reach	∙ Percentage of active clients who receive the EBP will be self-reported in provider surveys. Semi-structured qualitative interviews with providers will highlight reasons for stronger or weaker reach.	Provider surveys and qualitative interviews	4 m, 8 m, 12 m, 24 m
Adoption	∙ Percentage of providers who self-report practicing LGBTQ-affirmative CBT out of total who were trained. Semi-structured qualitative interviews with providers will highlight why and in what cases providers chose not to implement.	Provider surveys and qualitative interviews	baseline, 4 m, 8 m, 12 m, 24 m
Implementation	∙ Confidence with and use of LGBTQ-affirmative CBT skills will be measured using a scale adapted from the Counselor Activity Self-Efficacy scale [121] and Use of LGBTQ-affirmative CBT skills scales [57]. ∙ Modification of LGBTQ-affirmative CBT will be assessed by the Modification and Adaptation Checklist [122] and semi-structured qualitative interviews with providers.	Provider surveys and qualitative interviews	baseline, 4 m, 8 m, 12 m, 24 m
Maintenance	∙ Funding stability, environmental support, organizational capacity, and strategic planning will be measured by the Program Sustainability Assessment Tool [123] and semi-structured qualitative interviews with providers and directors.	Director surveys, provider surveys and qualitative interviews	4 m, 8 m, 12 m 24 m
Implementation determinants			
Inner setting	∙ Motivation for change, resources, and staff attributes will be measured by subscales of the Organizational Readiness for Change– Treatment Staff Version and Organizational Readiness for Change– Treatment Director Version [124].	Director surveys, provider surveys	baseline, 4 m, 24 m
Outer setting	∙ SGM Policy Climate (e.g., anti-LGBTQ laws/policies) will come from existing policy and attitudinal indices [35, 55, 125] linked to the municipality of each center. ∙ Community socioeconomic disadvantage and rurality (e.g., poverty, housing characteristics, unemployment, education) will come from the American Community Survey [126] and be linked to the county of each center. ∙ Mental health workforce characteristics (e.g., provider prevalence in each state, continuing education requirements of centers’ states) will derive from existing metrics [127]. ∙ Semi-structured qualitative interviews with providers and directors will inform assessment of the outer setting.	Secondary data, provider and director qualitative interviews	baseline
Intervention characteristics	∙ Directors’ and providers’ openness to adopting EBPs and perceived divergence between EBPs and current practice will come from the EBP Attitude Scale [128].	Director and provider surveys	baseline, 4 m, 24 m
Individual characteristics	∙ Provider, director, and client demographics (e.g., race/ethnicity, sexual orientation, gender) will be assessed via self-report questionnaires.	Provider, director, and client surveys	baseline, 4 m, 8 m, 12 m 24 m
Implementation process	∙ Implementation barriers and facilitators will come from interviews about staff planning, engagement, and execution, and other feedback on the implementation process [129]. ∙ Perceived appropriateness of the intervention and implementation strategy will be assessed with the Training and Adoption Barriers Scale [130] and the Intervention Appropriateness Measure [131]. ∙ Training engagement and satisfaction will be assessed via questionnaires designed for this study and an adapted version of the Client Satisfaction Questionnaire [132].	Director, provider, and supervisor qualitative interviews	4 m, 8 m, 12 m, 24 m
Implementation mechanisms			
Organization-level implementation targets	∙ Organizational norms of importance, expectations, and rewards of implementing LGBTQ-affirmative CBT will be measured with the Focus, Recognition, and Rewards subscales of the Implementation Climate Scale [133]. ∙ Organizational policies and communications about LGBTQ-affirmative CBT will be assessed with the Implementation Leadership Scale [134]. ∙ Supervision competency will be measured by the Supervision Evaluation and Supervisor Competence Scale [135].	Director, provider, and supervisor surveys	baseline, 4 m, 8 m, 12 m, 24 m
Provider-level implementation targets	∙ Provider LGBTQ-affirmative CBT competency will be measured using the LGBTQ-affirmative CBT Knowledge Scale [57]. ∙ Provider motivation to implement LGBTQ-affirmative CBT will be measured with the adapted Measure of Innovation-Specific Implementation Intentions [136]. ∙ Providers’ LGBTQ counseling competency will be assessed with the skills subscale of the Sexual Orientation Provider Competency Scale [137]. ∙ Providers’ familiarity and experience with case conceptualization will be assessed by a seven-item questionnaire developed for this study.	Provider surveys	baseline, 4 m, 8 m, 12 m, 24 m
	∙ Clients’ perceived therapeutic alliance will be measured with the Working Alliance Inventory [114]. ∙ Clients’ perceptions of therapist efficacy in LGBTQ-affirmative CBT skills will be assessed with the adapted Sexual Orientation Counselor Competency Scale [113].	Client surveys	baseline, 4 m, 8 m, 12 m, 24
Effectiveness outcomes			
Client mental health	∙ Depression and suicidality will be assessed with the Patient Health Questionnaire– 9 [88]. ∙ Anxiety will be assessed with the Generalized Anxiety Disorder Scale– 7 [89]. ∙ Substance use problems will be assessed with the Alcohol Use Disorder Identification Test– Consumption [90] and Drug Use Disorder Identification Test– Consumption [91].	Client surveys	baseline. 4 m, 8 m, 12 m, 24 m
LGBTQ-affirmative CBT targets	∙ Internalized stigma and LGBTQ identity affirmation will be measured using the Internalized Homonegativity and Identity Affirmation subscales of the Lesbian, Gay, Bisexual Identity Scale [92]. ∙ Social isolation will be assesed wtih the UCLA Loneliness Scale [138]. ∙ Emotion dysregulation will be assessed using the Difficulties in Emotion Regulation Scale– Short Form [139, 140].	Client surveys	baseline. 4 m, 8 m, 12 m, 24 m

### Implementation moderators

​​ In Aim 2, we will use the Consolidated Framework for Implementation Research (CFIR) [65, 66] to examine center-level implementation determinants at the inner setting (e.g., prior experience with EBPs), outer setting (e.g., local SGM policy climate, mental health workforce), intervention characteristics (e.g., attitude towards EBP), individual characteristics (e.g., staff turnover, client demographics), and implementation process (e.g., implementation barriers and facilitators) (see Table 1). Surveys and interviews with center directors and providers from baseline and 4-, 8-, 12-, and 24-month follow-up periods will provide data to inform whether CFIR-informed center-level determinants moderate these outcomes.

### Effectiveness outcomes

We will compare the three training strategies in terms of their impact on client mental health symptoms using reliable and valid brief symptom assessment measures. Primary effectiveness outcomes include symptoms of depression and suicidality [88] and secondary effectiveness outcomes include symptoms of anxiety [89] and substance use [90, 91]. We will also examine mediators of impact through the hypothesized target mechanisms of LGBTQ-affirmative CBT (e.g., internalized stigma) using reliable and valid assessments [92] using data collected at baseline and 4-, 8-, 12-, and 24-month follow-up periods (see Table 1).

### Implementation and effectiveness mediators

We will explore serial mediation of training strategies on implementation success and client mental health through organizational (e.g., shared norms of the importance of EBP) and provider (e.g., self-efficacy for EBP delivery) factors, drawing on theories of organizational [68, 69] and provider [70–73] change.

### Data collection, management, and analysis

Mental health provider, director, and local supervisor participants will complete baseline and follow-up surveys via Qualtrics. Client surveys will be administered and stored on REDCap. All participant consent, contact, tracking, and scheduling information will be recorded in REDCap.

#### Provider implementation fidelity outcome analyses

We will summarize provider characteristics (e.g., volunteer status, length of SGM service) overall and by implementation strategy and assess for baseline imbalances. We will then compare the implementation outcomes for the Materials Only condition vs. Direct Training vs. Local Supervision. The primary implementation outcome is provider implementation fidelity (assessed via simulated practice responses) at 8-month follow-up; secondary outcomes include 8-month provider self-efficacy, competency, familiarity, and use of LGBTQ-affirmative CBT. For primary and secondary outcomes, will use mixed effects models [93] with the identity link function to compare the effect of implementation strategies. The fixed effects include the time effect and the time × implementation strategies interaction terms; the random effects include center and within-provider repeated measures. We will use the robust score test to assess whether the interaction terms are significantly different across the three strategies, with a 0.05 overall Type I error rate, and a 0.025 error rate for each of the two primary strategy comparisons. For secondary outcomes, we will compare implementation strategies with a 0.05 Type I error rate. We will re-run these analyses with 12- and 24-month outcomes to determine implementation maintenance.

##### Sample size justification

Provider implementation fidelity serves as our primary outcome used to estimate sample size given its role in linking provider behavior to client mental health [84, 94–96]. We estimate that approximately six mental health providers will enroll per center, or about 540 providers total. Power was computed to test the null hypotheses of no difference between Materials Only and Direct Training and between Direct Training and Local Supervision in a cluster trial. For the primary implementation outcome, the change from baseline to 8-month follow-up was estimated as 0.14 for the Direct Training strategy and its variance was estimated as 0.0439 using data obtained from the waitlist-controlled trial [57]. Thus, the standardized change for the Direct Training strategy was estimated at 0.67. With a 0.025 Type I error rate, allowing for two primary comparisons between the three strategies, we will have 80% power to detect an effect of *d* = 0.34–0.46 on the 8-month change of the primary outcome, assuming a between-center ICC ≤ 0.1. In other words, we will have 80% power to detect the difference between Materials Only and Direct Training when the standardized change for Materials Only is ≤ 0.21–0.33, and we will have 80% power to detect the difference between Direct Training and Local Supervision when the standardized change for the Local Supervision strategy is ≥ 1.01–1.13.

#### Moderation analyses

To identify center- and provider-level determinants of implementation success, we will use stepwise regression [97], using 8-month simulated practice skills assessment of implementation fidelity as the primary outcome. Stepwise regression will iteratively add and remove each of the implementation determinants described previously and their interaction with implementation strategy to identify the subset of determinants with the minimal Akaike information criterion (AIC) [98]. We will re-run this analysis with each of the secondary implementation outcomes.

#### Effectiveness outcome analysis

We will summarize client characteristics — including age, gender, and race/ethnicity —overall and by implementation strategy and assess for baseline imbalances. We will then compare client mental health between Materials Only and Direct Training and between Direct Training and Local Supervision. The primary effectiveness outcome is the Patient Health Questionnaire– 9 at 8-month follow-up. In intent-to-treat analyses, we will use a mixed-effects model [93] and the identity link function to evaluate the effect of implementation strategies. The fixed effects include time and the time × implementation strategy interaction; random effects include center and within-client repeated measures. We will use the robust score test to assess whether the interaction terms are significantly different across and between the three strategies. We will use the inverse probability weighting method [99] to adjust for any selection bias due to loss to follow-up. For secondary outcomes (i.e., GAD-7, DUDIT-C, AUDIT-C, LGBIS, R-UCLA, and DERS-SF), we will also use mixed effects models and compare strategies with a 0.05 Type I error rate. In sensitivity analyses, to account for possible imbalances of client factors across the small number of clusters, we will adjust for these factors (e.g., age, gender, race/ethnicity) as well as center-level covariates (e.g., rurality, size) along with time and implementation strategies. We will use multiple imputation [100] to handle loss to follow-up and missing covariate data.

##### Sample size justification

We expect to collect mental health data from at least 52 clients in each of the 15 centers (*N* = 780). Power was computed to test the null hypotheses of no difference in outcomes between Materials Only and Direct Training and between Direct Training and Local Supervision in a cluster randomized trial. The standardized change in the primary outcomes (PHQ-9) over 8 months was estimated as 0.92 (est. = −0.79 [0.74]), for Local Supervision, the strategy used in a randomized controlled trial in which LGBTQ-affirmative CBT study therapists received ongoing supervision [48]. With a 0.05 Type I error rate, we have 80% power to detect an effect size of *d* = 0.33–0.37 for 8-month change, assuming a between-center ICC as large as 0.01 and loss to follow-up of 25%. In other words, we will have 80% power to detect the difference between Direct Training and Local Supervision when the standardized change for Direct Training is at most 0.55–0.59. Further, assuming that the standardized change in the outcome is 0.54 for Direct Training, we have 80% power to detect the difference between Materials Only and Direct Training when the standardized change in PHQ-9 is 0.18–0.26 or less for Materials Only.

#### Mediation analyses

We will assess client mental health as a function of our three implementation strategies as mediated through provider and organizational targets. We hypothesize that organizational (e.g., norms about LGBTQ-affirmative CBT) and provider (e.g., LGBTQ-affirmative CBT self-efficacy) targets will mediate the impact of implementation strategies on implementation success, and further on client mental health. We will use the difference approach [101] for multilevel mediation [102] of center-level implementation strategy on 8-month follow-up provider-level implementation fidelity through our hypothesized organizational and provider targets measured at 4-month follow-up. We will parse the implementation strategies’ total effect on the implementation outcome into direct and indirect (mediated) effects. Specially, we will fit a mixed-effects model of the implementation strategies on implementation outcome for the total effect and fit another mixed-effects model of the implementation strategies on implementation outcome plus center-level targets and provider-level targets. The fixed effects of the first model include the implementation strategies, baseline implementation fidelity, and baseline organizational and provider targets, and the second model replaces baseline organizational and provider targets with 4-month follow-up targets; random effects include center. The indirect effect is the difference between the estimated coefficients of implementation strategies between the two models; significance will be tested using bootstrapping [103]. We will use the same multilevel approach to test mediation of the association between implementation strategy and 12-month follow-up client-level PHQ-9. We will fit two mixed-effects models to estimate the total, direct and indirect (mediated) effects. The fixed effects of the first model include the implementation strategies, baseline PHQ-9, baseline implementation fidelity, and baseline organizational and provider targets, and in the second model, the baseline organizational and provider targets are replaced with 4-month follow-up targets and baseline implementation fidelity is replaced with 8-month follow-up implementation fidelity; the random effects include center and provider. We will re-run these analyses with 24-month follow-up PHQ-9 to determine maintenance of effects.

#### Qualitative analyses

Qualitative data will be longitudinally embedded into data collection during implementation to describe components of the implementation process and outcomes that cannot be answered quantitatively [104, 105]. We will use thematic analysis [106–108] and an abductive approach wherein we first generate codes inductively (e.g., emergent from the data) and then deductively (e.g., informed by the implementation frameworks). Inductive analysis will allow for unbiased description of the implementation process from center directors and supervisors and the creation of inductive codes. The deductive analysis will include integrating codes informed by CFIR and RE-AIM to capture the *why* and *how* of successful (or unsuccessful) implementation processes and outcomes (e.g., providers’ day-to-day perception of how supervision facilitated implementation, on-the-ground barriers to implementation not captured by the surveys). Sample sizes for the qualitative interviews will be sufficient to meet saturation (e.g., hearing it all and understanding it all for each implementation domain [109, 110]. All qualitative analyses will be conducted using Nvivo [111].

### Ethical considerations

This study has been approved by the Yale University Human Subjects Committee (2000035211) and has been registered at ClinicalTrials.gov (NCT05890404). The trial poses no greater than minimal risk to participants. The study team has taken every possible step to minimize breach of confidentiality. All research team members undergo privacy and confidentiality training, and participants will be assigned unique study identification numbers, keeping their survey and interview data separate from identifiable information. The consent documentation clearly delineates these risks and protections to participants. All participants (i.e., directors, providers, local supervisors, and clients) will provide consent electronically before completing their first survey.

A data and safety monitoring board (DSMB) will be convened annually to review study conduct, discuss potential safety and logistical concerns, and make recommendations regarding the resolution of these concerns and the status of the trial. This DSMB will be composed of experts in the field, and its members will not be affiliated with the study sponsor. Adverse events will be evaluated by PI Pachankis on a real-time basis and classified as serious (mild, moderate, severe) or non-serious, and appropriate reporting procedures will be followed for this. Important protocol modifications related to the safety of participants will be shared promptly with the National Institutes of Health and the Yale University Human Subjects Committee throughout the study. There are no plans to conduct independent audits or interim analyses of primary outcomes regarding the termination of the trial. 

### Dissemination

The final trial dataset will be available to all study investigators. Deidentified data for participants who have agreed to have their data shared will be submitted to the National Institutes of Mental Health Data Archive. In addition, the data and statistical code will be available by request to qualified researchers. To ensure that results of this research increase the likelihood that SGM individuals nationwide receive LGBTQ-affirmative CBT — one of the only EBPs developed to address SGM individuals’ mental health — and therefore have the strongest possible effect on SGM mental health disparities, we have planned for six months of dissemination activities. We will first present results to a community advisory board (CAB) of 12 directors, providers, and SGM community members to gain interpretive feedback and suggestions for dissemination activities. We will then summarize study results in an accessible research brief and accompanying webinar for all LGBTQ community center directors nationwide.

The brief and webinar content, which the CAB will review and shape, will identify the most effective implementation strategies for a variety of center-level conditions and specify barriers and facilitators of successful implementation so that center directors and providers can select implementation strategies accordingly. Alongside these results, we will instruct centers on how to access all implementation materials (i.e., for providers, supervisors, and clients) through our training platform. This dissemination phase will also allow us to explore centers’ maintenance or discontinuation of implementation strategies through exploratory interviews with directors and providers at centers representing diverse geographies, budgets, and staff and client makeup. These activities will inform future planning and needed supports on the part of community organizations, funders, and academic researchers to ensure that SGM continue to benefit from this and any future EBP capable of reaching SGM individuals across high-need, low-resource settings.

Authorship for manuscripts will be determined using the four criteria established by The International Committee of Medical Journal Editors [112]. Significant contribution for authorship includes contributions to the study conceptualization, study design, or data collection, analysis, or interpretation; drafting or critically reviewing the manuscript; approval of the final version of the manuscript; and the agreement to be held responsible for all aspects of the study and to assist in investigating any concerns about the study’s accuracy or integrity. An individual must meet each of these criteria to be considered as an author.

## Discussion

Although sexual and gender minority populations experience among the largest mental health disparities of any population, evidence-based mental health practice specifically targeting their distinct stressors and stress responses has been unavailable until recently. Now that LGBTQ-affirmative CBT has demonstrated efficacy across several trials and generated high demand, implementation science is needed to determine optimal nationwide implementation strategies. This type III hybrid effectiveness-implementation trial will identify the optimal means through which to implement this treatment in the US’s large network of LGBTQ community centers, thereby producing generalizable guidance for evidence-based practice (EBP) implementation across low-resource settings nationwide in which mental health disparity populations are likely to seek treatment.

This study represents the first to take advantage of the nationwide network of LGBTQ community centers, an innovative site for EBP implementation, to study the implementation of mental health EBP. National mental health networks are rare, yet the LGBTQ community created its own network of affirmative care settings to meet the needs of its members [53]. Coordinated by our partner CenterLink, this network of low-resource, frontline settings presents an innovative opportunity to broadly test the delivery of LGBTQ-affirmative CBT in these community settings. The study’s methods directly embrace features of low-resource mental healthcare settings (e.g., high volunteer staff, high turnover) to inform optimal implementation in such settings. First, by comparing three additive training levels, this study can determine the minimum training necessary in resource-constrained contexts. Second, recognizing that LGBTQ centers often rely on volunteers and trainees with high turnover, this study will follow providers over 24 months and assess relocation features for those who have moved (e.g., type of setting and continued fidelity to LGBTQ-affirmative CBT [113]). Third, this study will assess whether center leaders support LGBTQ-affirmative CBT as a way to overcome resource constraints (e.g., because it is time-limited and can attract funding). Finally, the local SGM policy context is constantly shifting, with impact on SGM clients’ mental health and centers’ ability to address it. The study team possesses expertise in studying policy impact on SGM mental health [8, 114, 115] and will assess the SGM policy context of each center as a moderator of implementation success [116]. This study advances implementation science using a rare opportunity to study EBP implementation in frontline settings in a key mental health disparity population.

## Supplementary Information

Supplementary Material 1.

## Data Availability

Data for this study will be made available at the study’s conclusion to qualified researchers upon request.

## Abbreviations

CBT: Cognitive behavioral therapy
CFIR: Consolidated Framework for Implementation Research
DSMB: Data and safety monitoring board
EBP: Evidence-based practice
SGM: Sexual and gender minority/minorities
