# Supplementary material for: Implementing LGBTQ-affirmative CBT: study protocol for an effectiveness-implementation trial at 90 LGBTQ community centers
Source: BMC Health Serv Res. 2025 Jul 21;25:962. doi: 10.1186/s12913-025-13136-3 (PMC12278521; doi:10.1186/s12913-025-13136-3)
Supplement: Supplementary file 1 — Supplementary Material 1. [file 12913_2025_13136_MOESM1_ESM.docx]

**PROVIDERS**

**CONSENT FOR PARTICIPATION IN A RESEARCH STUDY**

**YALE UNIVERSITY**

**Study Title:** Implementing sustainable evidence-based mental healthcare in low-resource community settings nationwide to advance mental health equity for sexual and gender minority individuals.

**Principal Investigator (the person who is responsible for this research):**

John Pachankis, Ph.D.
60 College Street
New Haven, CT 06510

**Research Study Summary:**

- We are asking you to join a research study.
- The purpose of this research study is to test different ways of training in evidence-based LGBTQ-affirmative cognitive-behavioral therapy (CBT) for mental health providers at LGBTQ community centers across the United States. Centers will be randomly assigned to receive one of three types of training strategies and we will compare how each training strategy influences knowledge and use of LGBTQ-affirmative CBT, as well as what center-level characteristics play a role in the uptake of this treatment.
- Providers at each center will be randomized to receive one or more of the following:
  - A digital suite of online self-guided learning materials, including step-by-step treatment materials, client handouts, and “how to” instructions for delivering the treatment.
  - 12 weekly 1-hour live training webinars on delivering LGBTQ-affirmative CBT led by expert trainers.
  - One year of ongoing (at least monthly) supervision in LGBTQ-affirmative CBT from a supervisor at your center.
- Regardless of your study condition described above, you will be asked to complete 30-minute surveys at the beginning of the study and 4, 8, 12, and 24 months later. You may also be asked to participate in a 1-hour interview with a member of our research team at each of the above timepoints.
- Your time involvement will vary based on the condition to which your center is assigned. If you are assigned to complete the digital suite of learning materials, this will take approximately 10-12 hours spread across several months. If you are assigned to also complete the weekly webinar training, you will spend about 12 hours in each of the 12 training sessions. If you are assigned to also receive supervision, this will take approximately another 12 hours.
- Surveys will take about 30 minutes each. Interviews will take about one hour each. You will be compensated for your time as described further below.
- There may be some risks from participating in this study. You may experience emotional discomfort as a result of survey questions, interviews, or discussions during trainings or supervision. There is also the possible risk of breach of confidentiality. Our research team’s strategies to mitigate these risks are described below.
- The study may have no benefits to you. You will be exposed to training resources for delivering LGBTQ-affirmative CBT, which may help you gain knowledge, competency, and motivation related to delivering LGBTQ-affirmative mental health care.
- Taking part in this study is your choice. You can choose to take part, or you can choose not to take part in this study. You also can change your mind at any time. Whatever choice you make will not have any effect on your relationship with your affiliated LGBTQ community center, our research team, or Yale University.
- If you are interested in learning more about the study, please continue reading, or have someone read to you, the rest of this document. Ask the study staff questions about anything you do not understand. Once you understand the study, we will ask you if you wish to participate; if so, you will have to check a box to indicate your consent on this form.

**Why is this study being offered to me?**

We are asking you to take part in a research study because you provide mental health services at, or receive referrals from, an LGBTQ community center in the United States. We are looking for at least 540 mental health providers from 90 centers to participate in this research study.

**Who is paying for the study?**

This study is funded by the National Institute of Mental Health.

**What is the study about?**

The purpose of this study is to test different forms of training in evidence-based LGBTQ-affirmative CBT for mental health providers at LGBTQ community centers across the United States. Providers at each center will receive one or more of the following: 1) a digital suite of online learning materials, 2) a weekly webinar training from our training team for 12 weeks, and/or 3) one year of supervision from a local supervisor at your center. We will compare how each training strategy influences your knowledge and use of LGBTQ-affirmative CBT, as well as what center-level characteristics play a role in this.

**What are you asking me to do and how long will it take?**

If you agree to take part, your participation in this study will involve 30-minute survey assessments at the beginning of the study and 4, 8, 12, and 24 months later. These surveys will ask for information about you as a provider and assess your knowledge and use of LGBTQ-affirmative CBT. You may also be asked to complete 1-hour interviews with a member of our research team at each timepoint. These interviews will discuss advantages and disadvantages of using LGBTQ-affirmative CBT in your practice. You may decline to participate in interviews without affecting your participation in the rest of the study.

Depending on which condition your center is assigned to, study activities will include one of the following:

- - Accessing a digital suite of online self-guided learning materials, including step-by-step treatment materials, client handouts, and “how to” instructions for delivering the treatment over four months
  - The above plus 12 weekly 1-hour live webinars on how to deliver LGBTQ-affirmative CBT led by expert trainers over four months
  - The above in addition monthly supervision in LGBTQ-affirmative CBT from an expert supervisor at your center over one year

Your involvement time will vary based on the condition to which your center is assigned. The digital suite will take approximately 10-12 hours to complete. The weekly webinar training will take 12 hours. Supervision will take about another 12 hours. Surveys will take about four hours in total over two years. Interviews will take up to five hours in total over two years. You will be compensated for your time completing surveys and interviews as described further below.

**Are there any risks from participating in this research?**

We do not expect any physical risks from taking part in this study. If you decide to take part in this study, you may experience emotional distress when completing survey or interview assessments on topics related to specific clients or organizational factors at your center. However, we believe these risks to be minimal. If you experience distress when completing the study questionnaires or interviews, you may choose to skip any question you do not wish to answer, take a break, or complete them later. To minimize the risk of experiencing emotional disturbance as a result of the surveys or training, you may also choose to discontinue participation at any time with no penalty.

There is also the possible risk of breach of confidentiality. The principal investigator and research team have been involved with numerous local, national, and international studies involving human subjects and have considerable experience implementing measures to protect confidentiality, and we therefore believe this risk to be low. All possible measures will be taken to protect your information provided to us through surveys and interviews, and your confidentiality will be respected. All data collected will identify you by a unique code such that your survey and interview responses cannot be connected with your individual records. All contact with our team will be made by research staff under explicit guidelines to preserve confidentiality when videoconferencing, telephoning, texting, or emailing information. You may provide alternative contact information (email, phone numbers, and mailing address) for compensation and study retention purposes. This information will be treated in the same confidential manner as all participant information. All materials with identifying information will be kept separate from study data in one password-protected electronic file on a secure server accessible only to members of the research team that requires dual-factor authentication to access.

If you report a clear intention to harm yourself or another person, we may need to contact a supportive person in your life or local emergency services. Only the minimal necessary identifying information will be provided to these people. Additionally, we will report suspected cases of child or elder abuse or neglect as required by law.

**How can the study possibly benefit me or others?**

You may benefit from taking part in this study. You will gain access to new training resources related to LGBTQ-affirmative CBT that may help you gain greater knowledge, competency, and motivation to deliver LGBTQ-affirmative mental health care. You may also receive direct training and/or supervision in LGBTQ-affirmative CBT delivery which may further facilitate your delivery of LGBTQ-affirmative practice.

Results of this study might also benefit society and LGBTQ people more generally and can potentially inform providers and agencies across the United States about the potential benefits of trainings to support LGBTQ individuals’ mental health.

**Are there any costs to participation?**

You will not have to pay for taking part in this study.

**Will I be paid for participation?**

You will be paid for taking part in this study. You will be compensated $20 for each survey assessment you complete (for a maximum total of $100). If you are asked to complete interviews, you will be compensated an additional $60 for each interview you complete (for a total of $300 and maximum total of $400). According to the rules of the Internal Revenue Service (IRS), payments for taking part in a study may be considered taxable income.

In addition, you may be eligible to receive one continuing education (CE) credit per completed training module, up to 12 CE credits.

**How will I be paid?**

You will be paid via Tango. Tango is an e-gift card service that allow you to choose from a variety of different vendors from which to receive a gift card. Tango gift cards would be delivered electronically to your email address.

**How will you keep my data safe and private?**

All of your responses will be held in strict confidence. Only the researchers involved in this study and those responsible for research oversight (such as representatives of the Yale University Human Research Protection Program, the Yale University Institutional Review Boards, and others) will have access to any information that could identify you. We will share such information with others if you agree to it or when we have to do it because U.S. or State law requires it. For example, we will tell somebody if we learn that you are hurting a child or an older person.

To best ensure confidentiality, all survey data will be collected via the secure Yale Qualtrics server. Similarly, all interview data will be collected via Yale’s secure Zoom platform. You will be assigned a unique identification number that will link your data collected across the study. A master link file will connect your contact information to your study identification number. The link file will be stored on Yale’s secure RedCap software, which is only accessible to approved study team members and requires dual-factor authentication to access. De-identified survey data will only be downloaded, organized, and stored on password-protected computers. All portable devices for this study will be encrypted.

All contact with our team will be made by research staff under explicit guidelines to preserve confidentiality when videoconferencing, telephoning, texting, or emailing information. You may provide alternative contact information (email, phone numbers, and mailing address) for compensation and study retention purposes. This information will be treated in a confidential manner. The database with your contact information will be deleted three years after completion of the study. All other data provided for this study, including any audio recordings that may be used for training or educational purposes, will be maintained securely for a minimum of three years.

When we publish the results of the research or talk about it in conferences, we will not use your name. If we want to use your name, we would ask you for your permission. We will also share information about you with other researchers for future research, but we will not use your name or other identifiers. We will not ask you for any additional permission.

**Certificate of Confidentiality**

This research is covered by a Certificate of Confidentiality from the National Institutes of Health. The researchers with this Certificate may not disclose or use information or documents that may identify you in any federal, state, or local civil, criminal, administrative, legislative, or other action, suit, or proceeding, or be used as evidence, for example, if there is a court subpoena, unless you have consented for this use. Information or documents protected by this Certificate cannot be disclosed to anyone else who is not connected with the research except, if there is a federal, state, or local law that requires disclosure (such as to report child abuse or communicable diseases but not for federal, state, or local civil, criminal, administrative, legislative, or other proceedings, see below); if you have consented to the disclosure, including for your medical treatment; or if it is used for other scientific research, as allowed by federal regulations protecting research subjects.

**What if I want to refuse or end participation before the study is over?**

Taking part in this study is your choice. You can choose to take part, or you can choose not to take part in this study. You also can change your mind at any time. Whatever choice you make will not have any effect on your relationship with your affiliated LGBTQ community center, our research team, or Yale University. You do not give up any of your legal rights by giving your consent to participate. If you decide to withdraw from the study, we will keep the information we have already collected from you to be used for data analysis purposes. However, you may also request that we delete your data completely.

**What will happen with my data if I stop participating?**

If you choose to stop participating in the study the data that has already been collected will be kept in order to maintain the integrity of the study, but no new information will be collected, and data will be kept deidentified. However, you may also elect to withdraw your data from the research.

**Investigator Interests**

Drs. Harkness, Pachankis, and Jackson are co-authors of manuals published by Oxford University Press in 2022, for which they receive royalties.

**Who should I contact if I have questions?**

Please feel free to ask about anything you don't understand.

If you have questions later or if you have a research-related problem, you can call the Principal Investigator at 646-429-9407 or email the research team at [lgbtqmentalhealth@yale.edu](mailto:lgbtqmentalhealth@yale.edu).

If you have questions about your rights as a research participant, or you have complaints about this research, you call the Yale Institutional Review Boards at (203) 785-4688 or email [hrpp@yale.edu](mailto:hrpp@yale.edu).

A description of this clinical trial will be available on <http://www.ClinicalTrials.gov>, as required by U.S. Law. This website will not include information that can identify you. At most, the Web site will include a summary of the results. You can search this Web site at any time.

**Documentation of Informed Consent**

This consent form is connected to your Study ID number; therefore, we do not need you to sign this document. By checking this box below, you are indicating that you read and understand this consent form and the information presented and that you agree to be in this study.

We will give you a digital copy of this consent form.

** I consent that I read and understand this consent form and information presented and agree to be in this study.**

We may want to contact you to inform you of future studies. Do we have your consent to keep your contact information on file?

|  **I agree to be contacted in the future regarding future studies from this research team.** |
| --- |

**DIRECTORS**

**CONSENT FOR PARTICIPATION IN A RESEARCH STUDY**

**YALE UNIVERSITY**

### **Study Title**

Implementing sustainable evidence-based mental healthcare in low-resource community settings nationwide to advance mental health equity for sexual and gender minority individuals.

**Principal Investigators (the person who is responsible for this research)**

John Pachankis, Ph.D., 60 College St., New Haven, CT 06520

**Research Study Summary:**

- The purpose of this research study is to test different ways of training in evidence-based LGBTQ-affirmative cognitive-behavioral therapy (CBT) for mental health providers at LGBTQ community centers across the United States. Centers will be randomly assigned to receive one of three types of training strategies and we will compare how each training strategy influences knowledge and use of LGBTQ-affirmative CBT, as well as assess what center-level characteristics play a role in the uptake of this treatment.
- Providers at each center will be randomized to receive one or more of the following:
  - A digital suite of online self-guided learning materials, including step-by-step treatment materials, client handouts, and “how to” instructions for delivering the treatment.
  - 12 weekly 1-hour live training webinars on delivering LGBTQ-affirmative CBT led by expert trainers.
  - One year of ongoing (at least monthly) supervision in LGBTQ-affirmative CBT from a supervisor at your center.
- Regardless of your center’s study condition described above, you will be asked to complete 30-minute surveys at the beginning of the study and 4, 8, 12, and 24 months later. You may also be asked to participate in a 1-hour interview with a member of our research team at each of the above timepoints.
- Surveys will take about 30 minutes each. Interviews will take about one hour each. You will be compensated for your time as described further below.
- There may be some risks from participating in this study. You may experience emotional discomfort as a result of survey questions, interviews, or discussions during trainings or supervision. There is also the possible risk of breach of confidentiality. Our research team’s strategies to mitigate these risks are described below.
- The study may have no benefits to you. Providers at your LGBTQ community center will receive training resources for delivering LGBTQ-affirmative CBT, which may help them gain knowledge, competency, and motivation related to delivering LGBTQ-affirmative mental health care.
- Taking part in this study is your choice. You can choose to take part, or you can choose not to take part in this study. You also can change your mind at any time. Given the importance to our research of having directors like you provide their perspectives, your data is necessary for us to make the best use of your centers’ involvement in this study. If you cannot participate, we therefore ask you to help us identify another director-level person at your center who can complete this survey.
- If you are interested in learning more about the study, please continue reading, or have someone read to you, the rest of this document. Ask the study staff questions about anything you do not understand. Once you understand the study, we will ask you if you wish to participate; if so, you will have to check a box to indicate your consent on this form.

**Why is this study being offered to me?**

We are asking you to take part in this research study because you are a clinical director or are in a similar leadership position at your LGBTQ community center. We are looking for 90 LGBTQ community center clinical directors from 90 centers to participate in this research study.

**Who is paying for the study?**

This study is funded by the National Institute of Mental Health.

**What is the study about?**

The purpose of this study is to test different forms of training in evidence-based LGBTQ-affirmative CBT for mental health providers at LGBTQ community centers across the United States. Centers will receive one or more of the following: 1) a digital suite of online learning materialseither: 1) a digital suite of online learning materials, 2) a weekly webinar training from our training team for 12 weeks, and/or 3) one year of supervision from a local supervisor at your center. We will compare how each training strategy influences your knowledge and use of LGBTQ-affirmative CBT, as well as what center-level characteristics play a role in this treatment.

**What are you asking me to do and how long will it take?**

If you agree to take part, your participation in this study will involve 30-minute survey assessments at the start of the study and 4, 8, 12, and 24 months later. These surveys will ask for information about you as a director/supervisor, your center, and the process of implementing LGBTQ-affirmative CBT. You may also be asked to complete 1-hour interviews with a member of our research team at each timepoint. These interviews will discuss advantages and disadvantages of using LGBTQ-affirmative CBT in your center. You may decline to participate in interviews without affecting your participation in the rest of the study.

**Are there any risks from participating in this research?**

We do not expect any physical risks from taking part in this study. If you decide to take part in this study, you may experience emotional distress when completing survey or interview assessments on topics related to specific clients or organizational factors at your center. However, we believe these risks to be minimal. If you experience mild distress when completing the study questionnaires or interviews, you may choose to skip any question you do not wish to answer, take a break, or complete them later. To minimize the risk of experiencing emotional disturbance as a result of the surveys or training, you may also choose to discontinue participation at any time. However, given the importance to our research of having directors like you provide their perspectives, your data is necessary for us to make the best use of your centers’ involvement in this study. If you cannot participate, we therefore ask you to help us identify another director-level person at your center who can complete this survey.

There is also the possible risk of breach of confidentiality. The principal investigator and research team have been involved with numerous local, national, and international studies involving human subjects and have considerable experience implementing measures to protect confidentiality, and we therefore believe this risk to be low. All possible measures will be taken to protect your information provided to us through surveys and interviews, and your confidentiality will be respected. All data collected will identify you by a unique code such that your survey and interview responses cannot be connected with your individual records. All contact with our team will be made by research staff under explicit guidelines to preserve confidentiality when videoconferencing, telephoning, texting, or emailing information. You may provide alternative contact information (email, phone numbers, and mailing address) for compensation and study retention purposes. This information will be treated in the same confidential manner as all participant information. All materials with identifying information will be kept separate from study data in one password-protected electronic file on a secure server accessible only to members of the research team that requires dual-factor authentication to access.

If you report a clear intention to harm yourself or another person, we may need to contact a supportive person in your life or local emergency services. Only the minimal necessary identifying information will be provided to these people. Additionally, we will report suspected cases of child or elder abuse or neglect as required by law.

**How can the study possibly benefit me or others?**

You or your center may benefit from taking part in this study. Your center will gain access to new training materials related to LGBTQ-affirmative CBT that may help providers at your center be able deliver LGBTQ-affirmative mental health care. You might find the surveys and interviews to be interesting and to provide helpful information about your center.

Results of this study might also benefit society and LGBTQ people more generally and can potentially inform providers and agencies across the United States about the potential benefits of trainings to support LGBTQ individuals’ mental health.

**Are there any costs to participation?**

There will be no cost to you if you choose to participate in this study.

**Will I be paid for participation?**

You will be paid for taking part in this study. You will be compensated $20 for each survey assessment you complete (for a maximum total of $100). If you are asked to complete interviews, you will be compensated an additional $60 for each interview you complete (for a total of $300 and maximum total of $400). According to the rules of the Internal Revenue Service (IRS), payments for taking part in a study may be considered taxable income.

**How will I be paid?**

You will be paid via Tango. Tango is an e-gift card service that allow the recipient to choose from a variety of different vendors from which to receive a gift card. Tango gift cards would be delivered electronically to your email address.

**How will you keep my data safe and private?**

All of your responses will be held in strict confidence. Only the researchers involved in this study and those responsible for research oversight (such as representatives of the Yale University Human Research Protection Program, the Yale University Institutional Review Boards, and others) will have access to any information that could identify you. We will share such information with others if you agree to it or when we have to do it because U.S. or State law requires it. For example, we will tell somebody if we learn that you are hurting a child or an older person.

To best ensure confidentiality, all survey data will be collected via the secure Yale Qualtrics server. Similarly, all interview data will be collected via Yale’s secure Zoom platform. You will be assigned a unique identification number that will link your data collected across the study. A master link file will connect your contact information to your study identification number. The link file will be stored on Yale’s secure RedCap software which is only accessible to approved study team members and requires dual-factor authentication to access. De-identified survey data will only be downloaded, organized, and stored on password-protected computers. All portable devices for this study will be encrypted.

All contact with our team will be made by research staff under explicit guidelines to preserve confidentiality when videoconferencing, telephoning, texting, or emailing information. You may provide alternative contact information (email, phone numbers, and mailing address) for compensation and study retention purposes. This information will be treated in a confidential manner. The database with your contact information will be deleted three years after completion of the study. All other data provided for this study, including any audio recordings that may be used for training or educational purposes, will be maintained securely for a minimum of three years.

When we publish the results of the research or talk about it in conferences, we will not use your name. If we want to use your name, we would ask you for your permission. We will also share information about you with other researchers for future research, but we will not use your name or other identifiers. We will not ask you for any additional permission.

**Certificate of Confidentiality**

This research is covered by a Certificate of Confidentiality from the National Institutes of Health. The researchers with this Certificate may not disclose or use information or documents that may identify you in any federal, state, or local civil, criminal, administrative, legislative, or other action, suit, or proceeding, or be used as evidence, for example, if there is a court subpoena, unless you have consented for this use. Information or documents protected by this Certificate cannot be disclosed to anyone else who is not connected with the research except, if there is a federal, state, or local law that requires disclosure (such as to report child abuse or communicable diseases but not for federal, state, or local civil, criminal, administrative, legislative, or other proceedings, see below); if you have consented to the disclosure, including for your medical treatment; or if it is used for other scientific research, as allowed by federal regulations protecting research subjects.

**What if I want to refuse or end participation before the study is over?**

Taking part in this study is your choice. You can choose to take part, or you can choose not to take part in this study. You also can change your mind at any time. Whatever choice you make will not have any effect on your relationship with Yale University. You do not give up any of your legal rights by giving your consent to participate. If you decide to withdraw from the study, we will keep the information we have already collected from you to be used for data analysis purposes. However, you may also request that we delete your data completely.

**What will happen with my data if I stop participating?**

If you choose to stop participating in the study, the data that has already been collected will be kept in order to maintain the integrity of the study, but no new information will be collected, and data will be kept de-identified. However, you may also elect to withdraw your data from the research.

**Who should I contact if I have questions?**

Please feel free to ask about anything you don't understand.

If you have questions later or if you have a research-related problem, you can call the Principal Investigator at 646-429-9407 or email the research team at [lgbtqmentalhealth@yale.edu](mailto:lgbtqmentalhealth@yale.edu).

If you have questions about your rights as a research participant, or you have complaints about this research, you call the Yale Institutional Review Boards at (203) 785-4688 or email [hrpp@yale.edu](mailto:hrpp@yale.edu).

A description of this clinical trial will be available on <http://www.ClinicalTrials.gov>, as required by U.S. Law. This Web site will not include information that can identify you. At most, the Web site will include a summary of the results. You can search this Web site at any time.

**Investigator Interests**

Drs Harkness, Pachankis, and Jackson are co-authors of manuals published by Oxford University Press in 2022, for which they receive royalties.

**Documentation of Informed Consent**

This consent form is connected to your Study ID number; therefore, we do not need you to sign this document. By checking this box below, you are indicating that you read and understand this consent form and the information presented and that you agree to be in this study.

We will give you a with a digital copy of this consent form.

 **I consent that I read and understand this consent form and information presented and agree to be in this study.**

We may want to contact you to inform you of future studies. Do we have your consent to keep your contact information on file?

|  **I agree to be contacted in the future regarding future studies from this research team.** |
| --- |
|  |

**CLIENTS**

**CONSENT FOR PARTICIPATION IN A RESEARCH STUDY**

**YALE UNIVERSITY**

**Study Title:** Implementing sustainable evidence-based mental healthcare in low-resource community settings nationwide to advance mental health equity for sexual and gender minority individuals

**Principal Investigator (the person who is responsible for this research):**

John Pachankis, Ph.D.
60 College Street
New Haven, CT 06510

**Research Study Summary:**

- We are asking you to join a research study.
- The purpose of this research study is to understand the effects of training mental health providers in LGBTQ-affirmative mental health practice. We are interested to know how your therapy might be affecting your mental health.
- Study activities will include taking a screener (1-min) and if eligible a brief (5-8 minutes) survey now and at subsequent timepoints (April 2025 and/or April 2026).
- We are interested in your experiences regardless of whether you are in therapy or not.
- Your total involvement will be about 10-30 minutes of time.
- The screener and surveys will ask about your background, mental health, and LGBTQ-related experiences.
- There may be some risks to participating in this study. You may experience emotional discomfort from the survey questions. There is also the possible risk of loss of confidentiality. The study team’s strategies to mitigate these risks are described below.
- The study may have no benefits to you. You may find it interesting and helpful to answer questions about your mental health. You also may feel good in knowing that your participation will help our research team better understand how to train mental health providers in LGBTQ-affirmative practice, which may help other members of the LGBTQ community in the future.
- Taking part in this study is your choice. You can choose to take part, or you can choose not to, take part in this study. You also can change your mind at any time. Whatever choice you make will have no effect on your relationship with your mental health provider, the center at which you receive services, or Yale University.
- If you are interested in learning more about the study, please continue reading, or have someone read to you, the rest of this document. Ask the study staff questions about anything you do not understand. Once you understand the study, we will ask you if you wish to participate; if so, you will have to sign this form.

**Why is this study being offered to me?**

We are asking you to take part in a research study because you are an LGBTQ client at an LGBTQ community center that is part of our study that looks at ways to enhance LGBTQ-affirmative therapy in LGBTQ community centers. We are looking for the clients at 15 LGBTQ+ community centers to be part of this research study.

**Who is paying for the study?**

This study is funded by the National Institutes of Mental Health.

**What is the study about?**

The purpose of this study is to better understand the effects of different ways of training mental health providers to deliver LGBTQ-affirmative mental health care at LGBTQ community centers. As part of this study, we will assess how this training impacts clients’ mental health.

**What are you asking me to do and how long will it take?**

If you agree to take part, your participation in this study will involve completing a screener (1 min) and a brief (about 5-8 minute) survey today. We will also recontact you in April 2024 and/or April 2026 to complete the brief (about 5-8 minute) survey again. Therefore, the study will take between 10-30 of your time over 1.5 years. The screener and surveys will ask about your background, mental health, and LGBTQ-related experiences. We are interested in your experiences regardless of whether you are in therapy or not.

**Are there any risks from participating in this research?**

We do not expect any physical risks from taking part in this study. If you decide to take part in this study, you may experience emotional discomfort as a result of answering survey questions about your anxiety, depression, and substance use. If you experience distress when completing the surveys, you may choose to skip any question you do not wish to answer, take a break, or complete the surveys at a later date.

There is also the possible risk of breach of confidentiality. The principal investigator and research team have been involved with numerous local, national, and international studies involving human subjects and have considerable experience implementing measures to protect confidentiality, and we therefore believe that there is a low risk of breach of confidentiality. All possible measures will be taken to protect information provided through surveys, and your confidentiality will be respected.

The only times when you will be asked to provide personally identifying information are at the bottom of this form, by signing your name to indicate consent, and when providing contact information (phone or email) to enter our raffle for survey completion. If you consent to participate in the study, you will be assigned a unique identification number. The pieces of identifying information you provide will be connected to your survey responses only through this identification number. A linking file with your identifying information and identification number will only be accessible to approved study team members and requires dual-factor authentication to access.

**How can the study possibly benefit me or others?**

You may benefit from taking part in this study. You may find it interesting and informative to complete surveys about your mental health and related experiences. You also may feel good knowing that your participation will help our team better understand how to train mental health providers in LGBTQ-affirmative practice, which may help other members of the LGBTQ community in the future.

**Are there any costs to participation?**

You will not have to pay for taking part in this study.

**Will I be paid for participation?**

You will be paid $10 for completing the survey. Payment will come in the form of a Tango gift card, which will allow you to choose from a variety of different vendors from which to receive a gift card. Tango gift cards would be delivered electronically to your email address. Once you complete your survey, you will also be asked if you would like to enter a raffle and enter your contact information. We will select 10 raffle winners, each of whom will receive a $100 e-gift card sent to their preferred contact address.

**How will you keep my data safe and private?**

All of your responses will be held in confidence. Only the researchers involved in this study and those responsible for research oversight (such as representatives of the Yale University Human Research Protection Program, the Yale University Institutional Review Boards, and other review boards) will have access to any information that could identify you that you provide.

To ensure that this survey is not accessed by people who do not meet the eligibility criteria, we will collect IP addresses during the online screening process. This IP address will be stored separately from your survey data. We will only use IP address to verify

To best ensure confidentiality, all survey data will be collected on Yale’s secure REDCap server. You will be assigned a unique identification number that will link your data collected across the study. A master link file will connect your contact information to your study identification number. The link file will be stored on Yale’s secure server RedCap software only accessible to the study team and requiring dual-factor authentication to access. De-identified survey data will only be downloaded, organized, and stored on password-protected computers. All portable devices used for this study will be encrypted. The database with contact information will be deleted three years after completion of the study. All other data provided for this study will be maintained securely for a minimum of three years.

We will only contact you to send you an e-gift card in the event you are selected for one of our raffles. This communication will be made under explicit guidelines to preserve confidentiality by our study staff, who are trained to maintain confidentiality.

When we publish the results of the research or talk about it in conferences, we will not use your name and will only report survey data in aggregate (combining across all participants). We may share information about you with other researchers for future research, but we will not use your name or other identifiers. We will not ask you for any additional permission.

We will collect your IP address via RedCap’s secure software. Your IP address will never be connected to your survey data, only the screener data so that we can ensure you are a real person, unique participant, indeed eligible for the study. Only senior study staff will have access to IP address.

**Certificate of Confidentiality**

This research is covered by a Certificate of Confidentiality from the National Institutes of Health. The researchers with this Certificate may not disclose or use information or documents that may identify you in any federal, state, or local civil, criminal, administrative, legislative, or other action, suit, or proceeding, or be used as evidence, for example, if there is a court subpoena, unless you have consented for this use. Information or documents protected by this Certificate cannot be disclosed to anyone else who is not connected with the research except, if there is a federal, state, or local law that requires disclosure (such as to report child abuse or communicable diseases but not for federal, state, or local civil, criminal, administrative, legislative, or other proceedings); if you have consented to the disclosure, including for your medical treatment; or if it is used for other scientific research, as allowed by federal regulations protecting research subjects.

**What if I want to refuse or end participation before the study is over?**

Taking part in this study is your choice. You can choose to take part, or you can choose not to take part, in this study. You also can change your mind at any time. Whatever choice you make will not have any effect on your relationship with your mental health provider, the center at which you receive services, or Yale University.

**Investigator Interests**

Drs. John Pachankis, Audrey Harkness, and Skyler Jackson are co-authors of books related to LGBTQ-affirmative mental health practice published by Oxford University Press in 2022, for which they receive royalties. These books are related to the practices being investigated in this study.

**Who should I contact if I have questions?**

Please feel free to ask about anything you don't understand.

If you have questions later or if you have a research-related problem, you can call the Principal Investigator at (646) 429-9407 or email the research team at [lgbtqmentalhealth@yale.edu](mailto:lgbtqmentalhealth@yale.edu).

If you have questions about your rights as a research participant, or you have complaints about this research, you call the Yale Institutional Review Boards at (203) 785-4688 or email [hrpp@yale.edu](mailto:hrpp@yale.edu).

A description of this clinical trial will be available on [*http://www.ClinicalTrials.gov*](http://www.ClinicalTrials.gov). This Website will not include information that can identify you. At most, the Website will include a summary of the results. You can search this Website at any time.

**Documentation of Informed Consent**

**The following is a list of key information pieces you have received about this research study. If you have any questions about any of these items, please contact the research team for more information before agreeing to participate. Please verify you understand the following items:**

- What the study is about.
- What I must do when I am in the study.
- The possible risks and benefits to me.
- Who to contact if I have questions.
- Any costs and payments.
- I can discontinue participating in the study at any time without penalty.
- All written and published information will be reported as group data with no reference to my name.
- I have been given the name of the researcher and others to contact.
- I have the right to ask any questions.

Your signature below indicates that you read and understand this consent form and the information presented and that you agree to be in this study.

We will give you a copy of this consent form.

| Participant Printed Name |  | Participant Signature |  | Date |
| --- | --- | --- | --- | --- |

**We may want to contact you to inform you of future studies. Do we have your consent to keep your contact information on file?**

- Yes, by indicating my consent here, I agree to be contacted in the future regarding future studies from this research team.
- No, I do not agree to be contacted in the future about studies from this research team.
